# Deeply located low-grade fibromyxoid sarcoma with FUS-CREB3L2 gene fusion in a 5-year-old boy with review of literature

**DOI:** 10.1186/s13000-014-0163-2

**Published:** 2014-09-03

**Authors:** Aiko Kurisaki-Arakawa, Yoshiyuki Suehara, Atsushi Arakawa, Tatsuya Takagi, Michiko Takahashi, Keiko Mitani, Kazuo Kaneko, Takashi Yao, Tsuyoshi Saito

**Affiliations:** Department of Human Pathology, Juntendo University School of Medicine, Bunkyo-ku, Tokyo Japan; Department of Orthopaedic Surgery, Juntendo University School of Medicine, 2-1-1, Hongo, Bunkyo-ku, Tokyo Japan

**Keywords:** Low-grade fibromyxoid sarcoma, FUS-CREB3L2, Fusion gene, Young patients

## Abstract

**Background:**

Low-grade fibromyxoid sarcoma (LGFMS) is a rare soft tissue tumor typically affecting young to middle-aged adults. Despite its otherwise benign histologic appearance and indolent nature, it can have fully malignant behavior, and recurrence and metastasis may occur even decades later.

**Case history:**

We report a case of LGFMS in the left lower leg of a 5-year-old Japanese boy. A magnetic resonance imaging (MRI) uncovered a well-demarcated intra-gastrocnemial tumor measuring 27 × 20 mm with a slightly high intensity on T1WI and heterogeneously high intensity on T2WI. Histologically, the tumor was composed of bland spindle-shaped cells with a whorled growth pattern. The tumor stroma was variably hyalinized and fibromyxoid with arcades of curvilinear capillaries and arterioles with associated perivascular fibrosis. Although LGFMS is known to affect children under 18 years of age, it is extremely rare in infants and children under 5 years of age. Despite the young age, this patient was accurately diagnosed by the typical histology and the detection of a FUS-CREB3L2 gene fusion.

**Conclusion:**

Although LGFMS in children tends to be located superficially, this case presented with an intramuscular tumor in the region of the gastrocnemius. To the best of our knowledge, this is the first case of deep LGFMS arising in a child younger than 5 years of age. The patient is still alive with no evidence of the disease 4 months after diagnosis.

**Virtual Slides:**

The virtual slide(s) for this article can be found here: http://www.diagnosticpathology.diagnomx.eu/vs/13000_2014_163

## Background

Low-grade fibromyxoid sarcoma (LGFMS) is a rare soft tissue tumor that typically affects young to middle-aged adults [1,2]. The median age of onset for LGFMS is 34 years [3], although patients of any age can be affected, and 13-19% of cases occur in patients 18 years and younger [3,4]. However, LGFMS is rare in children during the first 5 years of life [2]. To the best of our knowledge, the youngest case of LGFMS is a tumor arising from the cheek in a 22-month-old girl [4]. Histologically, LGFMS is composed of bland spindle-shaped cells in a whorled growth pattern, arranged in alternating myxoid and collagenized areas along with curvilinear capillaries and characteristic arterioles with perivascular fibrosis. Although most of the tumor cells show benign histological appearance, approximately 10% of the tumor may have scattered large, hyperchromatic and pleomorphic nuclei [1]. Furthermore, approximately 40% of cases have focal areas of hypocellular collagen cores rimmed by epithelioid fibroblasts, referred to as collagen pseudo-rosettes. Cases with prominent collagen pseudo-rosettes are referred to as hyalinizing spindle cell tumor with giant rosettes [5].

In spite of its otherwise benign histologic appearance and indolent progression, many cases of the LGFMS may recur or metastasize decades later, mainly to the lung. Therefore, it is important to make a correct diagnosis and provide long-term follow-up for LGFMS patients. However, a diagnosis of LGFMS is often difficult because of a small biopsy specimen that can lead to a misdiagnosis of malignant tumors as benign or as tumor-like lesions including nodular fasciitis, schwannoma, desmoid-type fibromatosis, neurofibroma, and myxofibrosarcoma [6–8].

Although, LGFMS is negative for most immunohistochemical markers, a recent study identified up-regulation of the mucin 4 (MUC4) gene in LGFMS compared with histologically similar tumors and lesions [8,9], and MUC4 immunostaining was a sensitive and specific marker of LGFMS in the appropriate morphologic context [2,5]. Additionally, it has been described that claudin-1, which was associated with tight junction protein, and epithelial membrane antigen (EMA) were negative for LGFMS [10] with exceptional positivity for LGFMS with perineuriomatous component [11]. Perineurioma is a benign soft tissue tumor, derived from perineural cells which differ from other mesenchymal cells by virtue of their formation of tight junctions [10].

Several recent studies demonstrated that greater than 90% of LGFMS have a balanced chromosomal translocation t (7;16) (q32-34;p11) leading to the fusion of the FUS and CREB3L2, while a minority of cases have a t(11;16) (p11;p11) translocation leading to the fusion of the FUS and CREB3L1 [1,2,5–7,12,13]. In one report, a small number of LGFMS cases contained EWSR-CREB3L1 gene fusions [5].

We report a case of a 5-year-old Japanese boy with deeply located LGFMS in the lateral aspect of the lower leg. The diagnosis of LGFMS was confirmed by the presence of the characteristic FUS-CREB3L2 gene fusion using reverse transcription polymerase chain reaction (RT-PCR) from a formalin-fixed, paraffin-embedded (FFPE) biopsy specimen.

## Case presentation

**Figure 1:**
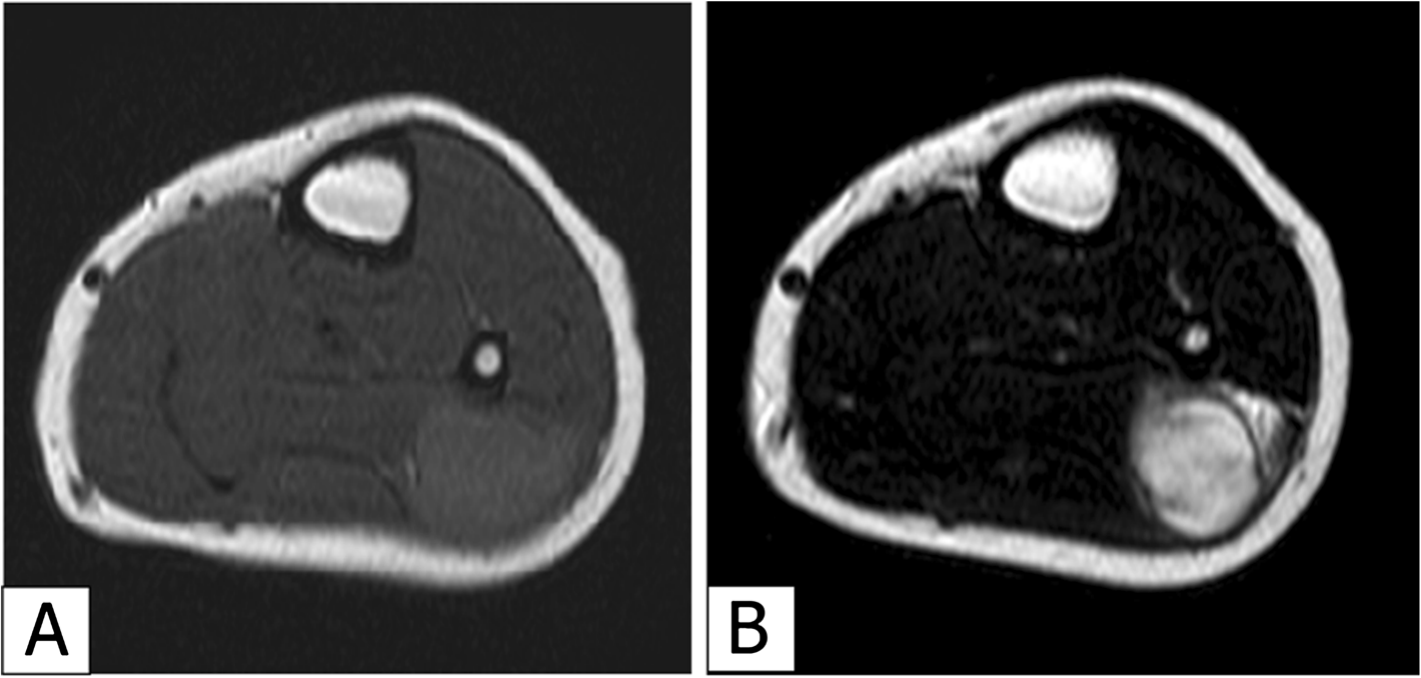
**MRI of lower left leg of the 5-year-old patient.** An axial view MRI revealed a well-defined mass measuring 27 × 20 mm in the lateral aspect of the gastrocnemius. The mass had slightly high intensity compared to the skeletal muscle on T1WI **(A)** and a heterogeneously high intensity on T2WI **(B)**.

**Figure 2:**
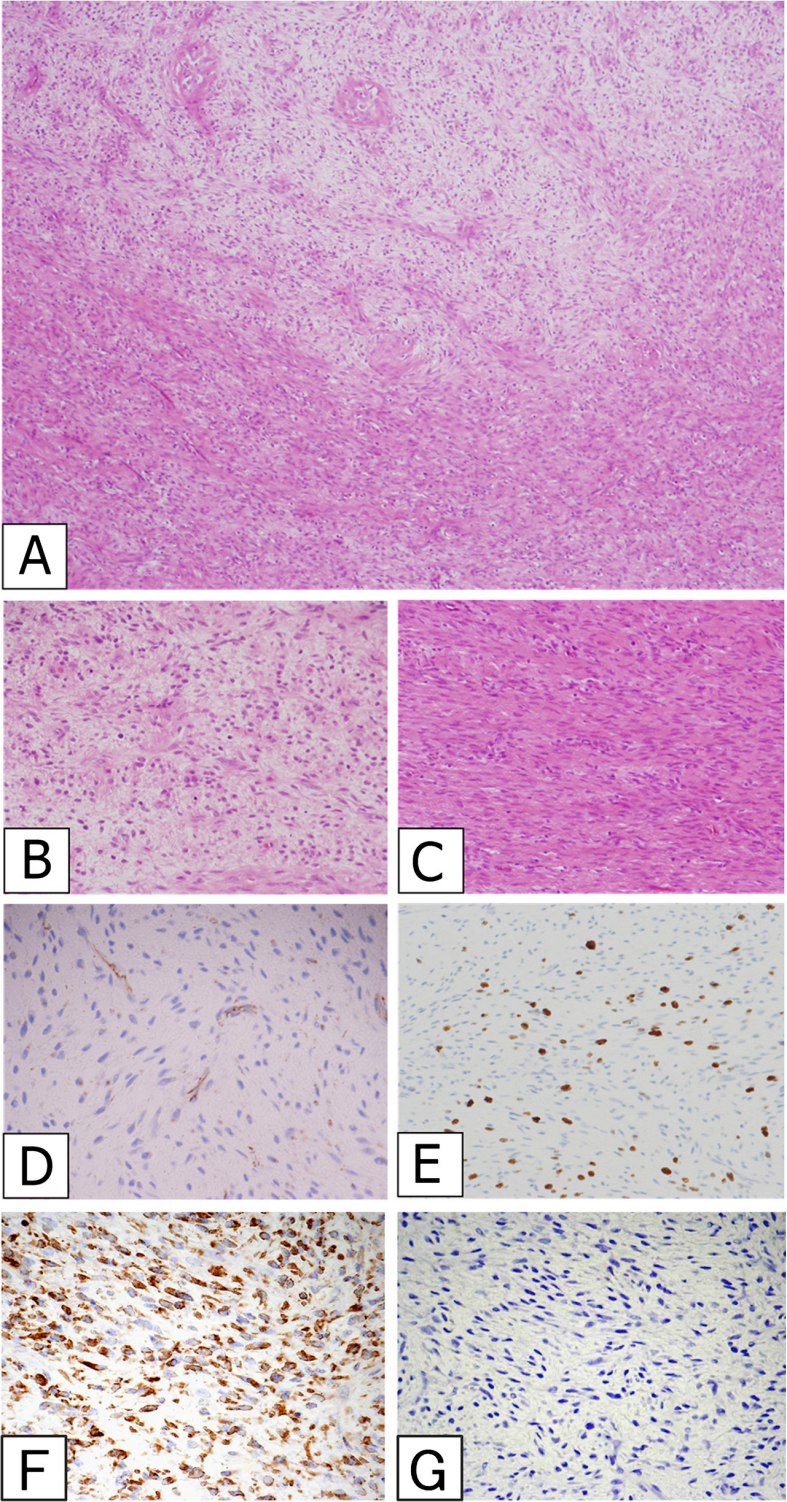
**Tumor Histology.** An admixture of myxoid and fibrous areas was observed on H&E staining **(A)**. Higher magnification views of myxoid **(B)** and fibrous **(C)** components of the tumor. A whorled growth pattern with bland spindle-shaped tumor cells was observed. Immunoreactivity for nuclear β-catenin was absent **(D)**. Ki67 staining was present with a proliferative index of approximately 20% **(E)**. Tumor cells showed diffuse and strong immunoreactivity for MUC4 **(F)**. Tumor was negative for claudin-1 **(G)**.

A 5-year-old Japanese boy was referred to our hospital with a painless mass in the lateral aspect of the left lower leg. An MRI revealed a well-demarcated intra-gastrocnemial tumor measuring 20 × 27 mm with slightly high intensity on T1WI and heterogeneously high intensity on T2WI (Figures 1A and 1B). Histopathologically, the tumor was composed of bland spindle-shaped cells with a whorled growth pattern on the biopsy specimen. The tumor stroma was variably hyalinized and fibromyxoid with arcades of curvilinear capillaries and arterioles with perivascular fibrosis (Figure 2A-2C). The tumor showed no nuclear pleomorphism, high cellularity, or necrosis, and had few mitoses. Differential diagnosis on conventional H&E staining included desmoid-type fibromatosis, schwannoma, and LGFMS. Immunohistochemically, the tumor cells were negative for S-100 protein and nuclear staining of beta-catenin, typically present in desmoid-type fibromatosis. The Ki67 proliferation index was approximately 20% (Figure 2D and 2E). Moreover, we performed immunohistochemical staining for MUC4 (sc-53945, Santa-Cruz Biotechnology, Dallas, TX, 1:100, mouse monoclonal), claudin-1 (Product# LS-B6327, LifeSpan Biosciences, Seattle, WA, 1:200, rabbit polyclonal) and EMA (E29, DAKO, Grostrup, Denmark, 1:100, mouse, monoclonal) on this case. Tumor cells showed diffuse, strong cytoplasmic immunoreactivity for MUC4. On the other hand, the tumor did not show immunoreactivity for claudin-1 and EMA in line with the absence of perineuriomatous component (Figure 2F and 2G).

**Figure 3:**
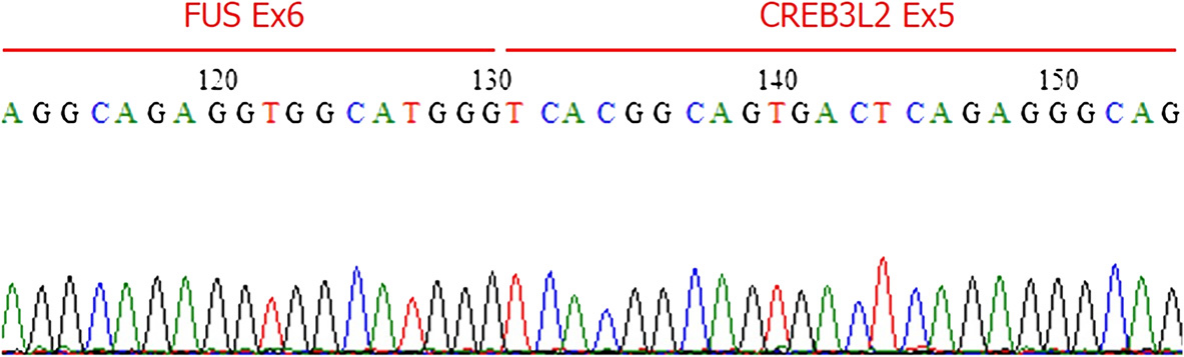
**Detection of a FUS-CREB3L2 fusion in the LGFMS tumor.** RT-PCR on FFPE-derived RNA was performed. DNA sequencing revealed a fusion between FUS exon 6 and CREB3L2 exon 5.

**Figure 4:**
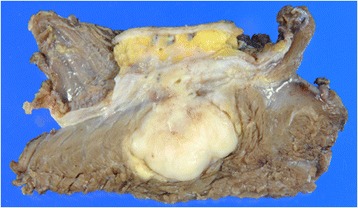
**Gross morphology of LGFMS from the 5-year-old patient.** The resected surface of intramuscular tumor is tan to gray colored and has a glistening appearance. The tumor is located in the subcutis and skeletal muscle throughout the fascia.

To determine if the FUS-CREB3L1 and FUS-CREB3L2 fusion genes were present in the tumor, we performed RT-PCR from FFPE tumor tissue. Briefly, five 10-μm thick paraffin sections were cut from the paraffin-embedded block. RNA was isolated using the RNeasy FFPE kit (QIAGEN, Hilden, Germany). Purified RNA was reverse transcribed to cDNA using the Superscript first-strand synthesis system for RT-PCR (Invitrogen, CA, USA). The primer sequences used for the amplification in this study were described previously [14]. The PCR product was separated on a 2% agarose gel, and a PCR product of the appropriate size was cut from the gel and sequenced. Sequencing confirmed a gene fusion between exon 6 of the FUS and exon 5 of CREB3L2 (Figure 3). Despite the young age of the patient, a diagnosis of LGFMS was confirmed, and the patient subsequently underwent wide resection at the affected area. Macroscopically, the resected surface of the surgical specimen was gray with a glistening appearance (Figure 4). Pathological analysis of the resected tumor confirmed the LGFMS histology observed in the biopsy specimen. Four months after diagnosis, the patient is alive with no evidence of the disease.

## Discussion

The importance of molecular pathological diagnosis is increasing in the clinical pathology, especially in the field of soft tissue sarcomas, because the definite diagnosis made by the molecular pathology such as RT-PCR and specific genetic testing sometimes leads to the application of the tumor specific therapy [15–17]. Recently, LGFMS was shown to be associated with gene fusions involving CREB3-family genes that encode members of the basic leucine zipper family of transcription factors [2]. The majority of LGFMS cases (95%) have a fusion of the FUS-CREB3L2, and a minority of cases (5%) have a FUS-CREB3L1 gene fusion. In addition, a small number of cases have EWSR-CREB3L1 gene fusions [5]. Because of its typical histology and the presence of a FUS-CREB3L2 fusion, we were able to correctly diagnose LGFMS, despite the atypical age of the patient. LGFMS can affect patients of all ages, and 13-19% of the cases occur in patients younger than 18 years of age [3,4]. Nonetheless, desmoid-type fibromatosis would be the most important differential diagnosis in this deeply located tumor. Desmoid-type fibromatosis is a locally aggressive infiltrative intra-muscular tumor, in spite of benign histologic feature. Frequent recurrences can be clinically observed even after wide resection. A “wait-and-see” strategy is, at the present time, preferred in case of asymptomatic or non-progressive disease, especially for the patients of young age. Medical treatment such as cyclooxygenase-2 selective inhibitor is another choice for the patients with desmoid-type fibromatosis [18].

LGFMS is extremely rare in children younger than 5 years of age [2], and we found only 34 LGFMS cases in children under 15 years of age reported in English literatures. The clinical, histological, and genetic information of the previously reported cases are summarized in Table 1 [1,2,4–7,12,13,19–27]. Nine cases up to 5 years of age were found including the present case. In our review of LGFMS in infants and young adolescents, the male to female ratio was 7:2 in infants (0-5 years of age) and 8:5 in young adolescents (6-15 years of age). In adult cases, the male to female ratio was reported to be either equal [7] or 3:1 [28]. The greatest mean tumor diameter was 3.7 cm in both infants and young adolescents, and this was smaller than that found in adult cases (mean 7.7 cm) [4]. LGFMS tends to be located superficially in young patients, whereas deep LGFMS accounts for the majority of adult cases [1,7]. Superficial LGFMS was reported to account for no more than 10% of LGFMS cases in all ages [4]; however, it was found in 73% of the patients 15 years and younger in our literature review, in line with the previous finding [22]. Particularly, in patients 5 years of age or younger in which information for the depth of the tumor was available, all cases were located superficially, and our case was the only one that was deeply located.

**Table 1:** **Literature review of LGFMS of children (≦15 y. o.)**

**Case**	**Age**	**Sex**	**Site**	**Location**	**Size**	**Genetic abnormality**	**Characteristic feature**	**Recurrence**	**Reference**
1	10	M	back	subcutis		FUS gene rearrangement (+) by FISH	pleomorphism (+)		[1]
2	8	M	pelvis	subfascial	φ80mm	(−)		local recurrence and intra abdominal meta 8 mos later	[6]
3	14	M	brachium	subfascial	φ50mm	(−)			[6]
4	14	M	brachium	subfascial	φ30mm	FUS gene rearrangement (+) by FISH			[6]
5	6	M	Lower leg	subcutis	φ10mm	EWSR1 (Ex9)/ CREB3L1 (Ex5) fusion gene by RT-PCR		(−)	[5]
6	13	M	popliteal fossa	deep		FUS/ CREB3L1 fusion gene by array-CGH		(−)	[12]
7	3	M	thigh	subcutis	17 × 16 × 12 mm	FUS gene rearrangement (+) by FISH and	unusual histology+	recurrence 1 year later	[2]
						other chromosomal translocations (7q34, 10q11.2, 16p11.2)	calponin (+)		
8	5	M	buttock	subcutis	29 × 17 × 25 mm	FUS gene rearrangement (+) by FISH		(−)	[7]
9	6	F	inguinal area		φ35mm			recurrence 5 times, meta for lung 39 years later	[19]
10	9	M	neck					recurrence 7 times, meta 16 years later	[19]
11	6	M	deltoid		φ33mm			(−)	[19]
12	10	F	leg				with giant rosettes		[20]
13	13	F	back					(−) at 36mos	[20]
14	1.8	M	cheek		φ80mm			(−) 6mo	[4]
15	13	F	arm		φ30mm	FUS (Ex6)/ CREB3L2 (Ex5) fusion gene by RT-PCR		(−) at 34mos	[21]
16	11	M	axilla		φ60mm	FUS (Ex6)/ CREB3L2 (Ex5) fusion gene by RT-PCR			[21]
17	15	M	thorax		φ30mm	FUS (Ex6)/ CREB3L1 (Ex5) fusion gene by RT-PCR			[21]
18	13	M	foot		φ60mm	FUS (Ex6)/ CREB3L2 (Ex5) fusion gene by RT-PCR		pleural metastasis at 180, 186, and 203mos,	[21]
								pleuropulmonary metastasis at 228 mos	
19	13	F	abdominal wall	intramuscular		FUS (Ex6)/ CREB3L2 (Ex5) fusion gene by RT-PCR			[13]
20	2	F	groin/vulva	superficial	φ20mm			(−) at 84mos	[22]
21	3	F	knee/popliteal fossa	superficial	φ42mm			(−) at 60mos	[22]
22	5	M	knee	superficial	φ40mm			(−) at 48mos	[22]
23	6	M		superficial	φ20mm			lost to follow up	[22]
24	8	M	back	superficial	φ30mm			local recurrence at 16mos, then lost to follow up	[22]
25	10	F	lower leg	superficial	φ16mm			(−) at 48mos	[22]
26	13	M	fore arm	superficial				(−) at 60mos	[22]
27	13	F	thigh			FUS (Ex6)-insertion-CREB3L2 (Ex5) fusion gene by RT-PCR		local recurrence at 156, 192, 228, 252, 264,	[23]
								and 276 mos	
28	12	F	buttock		φ30mm	FUS (Ex6)/ CREB3L2 (Ex5) fusion gene by RT-PCR		lost to follow up	[23]
29	12	M	foot		φ30mm	FUS (Ex6)/ CREB3L2 (Ex5) fusion gene by RT-PCR		local recurrence at 19mos	[23]
30	4	M	paravertebral		90 × 80 × 60 mm		with giant rosettes	local recurrence at 3mos	[24]
31	13	M	buttock	superficial				local recurrence at 24, 36, 60mos	[25]
32	11	F	shoulder	deep	φ45mm			(−) 34mos	[25]
33	14	F	inflaclavicular		φ30mm			(−) 58mos	[26]
34	4	M	thigh		φ30mm			local recurrence at 6, 36, and 84mos	[27]
								lung metastasis at 36, 84, and 98mos	
this case	5	M	Lower leg	subfascial	φ26mm	FUS (Ex6)/ CREB3L2 (Ex5) fusion gene by RT-PCR		(−) at 4 mos	

Empty spaces: Data not available.

Considering both the smaller size and superficial location of LGFMS arising in correspondingly smaller infants and young adolescents, LGFMS in children might be noticed before becoming larger and invading into the fascia and skeletal muscles. Metastasis/local recurrence rates were 13%/38% in infants and 24%/41% in young adolescents, respectively, in our literature review. These rates are not significantly different from those previously reported in adult LGFMS [19]. In addition, no differences in gene fusion events were noted between infants and young adolescents with LGFMS.

Finally, we report a rare case of a 5-year-old Japanese boy with deeply located LGFMS in the lateral aspect of the lower leg. The superficial location of LGFMS in pediatric patients might be a reason for the relatively small tumor size in juvenile populations. Furthermore, superficial tumors are easily palpable and readily recognized by the patients and their parents.

## Conclusions

Wide resection of the tumor in the limb reduces the recurrence rate, but also affecting the future functional outcome, especially in the childhood. Recent advance in molecular pathology is helpful to distinguish specific soft tissue tumors from their histologic mimics. It is important to avoid misdiagnosis and provide appropriate treatment with a help of molecular diagnosis.

## Consent

Written informed consent was obtained from the parents of the patient for the publication of this report and accompanying images. The copy of the written consent is available for review by the Editor-in-Chief of this Journal.

## References

[1] Sedrak MP, Parker DC, Gardner JM (2014). Low-grade fibromyxoid sarcoma with nuclear pleomorphism arising in the subcutis of a child. J Cutan Pathol.

[2] Dobin SM, Malone VS, Lopez L, Donner LR (2013). Unusual histologic variant of a low-grade fibromyxoid sarcoma in a 3-year-old boy with complex chromosomal translocations involving 7q34, 10q11.2, and 16p11.2 and rearrangement of the FUS gene. Pediatr Dev Pathol.

[3] Folpe AL, Lane KL, Paull G, Weiss SW (2000). Low-grade fibromyxoid sarcoma and hyalinizing spindle cell tumor with giant rosettes: a clinicopathologic study of 73 cases supporting their identity and assessing the impact of high-grade areas. Am J Surg Pathol.

[4] Tang Z, Zhou ZH, Lv CT, Qin LY, Wang Y, Tian G, Luo XL, Zhu Q, Xu XG (2010). Low-grade fibromyxoid sarcoma: clinical study and case report. J Oral Maxillofac Surg.

[5] Lau PP, Lui PC, Lau GT, Yau DT, Cheung ET, Chan JK (2013). EWSR1-CREB3L1 gene fusion: a novel alternative molecular aberration of low-grade fibromyxoid sarcoma. Am J Surg Pathol.

[6] Maretty-Nielsen K, Baerentzen S, Keller J, Dyrop HB, Safwat A (2013). Low-grade fibromyxoid sarcoma: Incidence, treatment strategy of metastases, and clinical significance of the FUS gene. Sarcoma.

[7] Menon S, Krivanek M, Cohen R (2012). Low-grade fibromyxoid sarcoma, a deceptively benign tumor in a 5-year-old child. Pediatr Surg Int.

[8] Doyle LA, Möller E, Dal Cin P, Fletcher CD, Mertens F, Hornick JL (2011). MUC4 is a highlysensitive and specific marker for low-grade fibromyxoid sarcoma. Am J Surg Pathol.

[9] Möller E, Hornick JL, Magnusson L, Veerla S, Domanski HA, Mertens F (2011). FUS-CREB3L2/L1-positive sarcomas show a specific gene expression profile with upregulation of CD24 and FOXL1. Clin Cancer Res.

[10] Folpe AL, Billings SD, McKenney JK, Walsh SV, Nusrat A, Weiss SW (2002). Expression of claudin-1, a recently descrived tight junction-associated protein, distinguishes soft tissue perineurioma from potential mimics. Am J Surg Pathol.

[11] Thway K, Fisher C, Debic-Rychter M, Calonje E (2009). Claudin-1 is expressed in perineuroma-like low-grade fibromyxoid sarcoma. Hum Pathol.

[12] Odem JL, Oroszi G, Bernreuter K, Grammatopoulou V, Lauer SR, Greenberg DD, Vogler CA, Batanian JR (2013). Deceptively benign low-grade fibromyxoid sarcoma: array-comparative genomic hybridization decodes the diagnosis. Hum Pathol.

[13] Panagopoulos I, Storlazzi CT, Fletcher CD, Fletcher JA, Nascimento A, Domanski HA, Wejde J, Brosjö O, Rydholm A, Isaksson M, Mandahl N, Mertens F (2004). The chimeric FUS/CREB3l2 gene is specific for low-grade fibromyxoid sarcoma. Genes Chromosomes Cancer.

[14] Matsuyama A, Hisaoka M, Shimajiri S, Hayashi T, Imamura T, Ishida T, Fukunaga M, Fukuhara T, Minato H, Nakajima T, Yonezawa S, Kuroda M, Yamasaki F, Toyoshima S, Hashimoto H (2006). Molecular detection of FUS-CREB3L2 fusion transcripts in low-grade fibromyxoid sarcoma using formalin-fixed, paraffin-embedded tissue specimens. Am J Surg Pathol.

[15] Li J, Yin WH, Takeuchi K, Guan H, Huang YH, Chan JK (2013). Inflammatory myofibroblastic tumor with RANBP2 and ALK gene rearrangement: a report of two cases and literature review. Diagn Pathol.

[16] Lin XY, Wang Y, Yu JH, Liu Y, Wang L, Li QC, Wang EH (2013). Sclerosing rhabdomyosarcoma presenting in the masseter muscle: a case report. Diagn Pathol.

[17] Martorell M, Ortiz CM, Garcia JA (2010). Testicular fusocellular rhabdomyosarcoma as a metastasis of elbow sclerosing rhabdomyosarcoma: a clinicopathologic, immunohistochemical and molecular study of one case. Diagn Pathol.

[18] Mignemi NA, Itani DM, Fasig JH, Keedy VL, Hande KR, Whited BW, Homlar KC, Correa H, Coffin CM, Black JO, Yi Y, Halpern JL, Holt GE, Schwartz HS, Schoenecker JG, Cates JM (2012). Signal transduction pathway analysis in desmoid-type fibromatosis: transforming growth factor-β, COX2 and sex steroid receptors. Cancer Sci.

[19] Evans HL (2011). Low-grade fibromyxoid sarcoma: a clinicopathologic study of 33 cases with long-term follow-up. Am J Surg Pathol.

[20] Rekhi B, Deshmukh M, Jambhekar NA (2011). Low-grade fibromyxoid sarcoma: a clinicopathologic study of 18 cases, including histopathologic relationship with sclerosing epithelioid fibrosarcoma in a subset of cases. Ann Diagn Pathol.

[21] Guillou L, Benhattar J, Gengler C, Gallagher G, Ranchère-Vince D, Collin F, Terrier P, Terrier-Lacombe MJ, Leroux A, Marquès B, Aubain Somerhausen Nde S, Keslair F, Pedeutour F, Coindre JM (2007). Translocation-positive low-grade fibromyxoid sarcoma: clinicopathologic and molecular analysis of a series expanding the morphologic spectrum and suggesting potential relationship to sclerosing epithelioid fibrosarcoma: a study from the French Sarcoma Group. Am J Surg Pathol.

[22] Billings SD, Giblen G, Fanburg-Smith JC (2005). Superficial low-grade fibromyxoid sarcoma (Evans Tumor): a clinicopathologic analysis of 19 cases with a unique observation in the pediatric Population. Am J Surg Pathol.

[23] Mertens F, Fletcher CD, Antonescu CR, Coindre JM, Colecchia M, Domanski HA, Downs-Kelly E, Fisher C, Goldblum JR, Guillou L, Reid R, Rosai J, Sciot R, Mandahl N, Panagopoulos I (2005). Clinicopathologic and molecular genetic characterization of low-grade fibromyxoid sarcoma, and cloning of a novel FUS/CREB3L1 fusion gene. Lab Invest.

[24] Rando G, Buonuomo V, D’Urzo C, Vecchio F, Caldarelli M, Pintus C (2005). Fibromyxoid sarcoma in a 4-year-old boy: case report and review of the literature. Pediatr Surg Int.

[25] Oda Y, Takahira T, Kawaguchi K, Yamamoto H, Tamiya S, Matsuda S, Tanaka K, Iwamoto Y, Tsuneyoshi M (2004). Low-grade fibromyxoid sarcoma versus low-grade myxofibrosarcoma in the extremities and trunk. A comparison of clinicopathological and immunohistochemical features. Histopathology.

[26] Lane KL, Shannon RJ, Weiss SW (1997). Hyalinizing spindle cell tumor with giant rosettes: a distinctive tumor closely resembling low-grade fibromyxoid sarcoma. Am J Surg Pathol.

[27] Canpolat C, Evans HL, Corpron C, Andrassy RJ, Chan K, Eifel P, Elidemir O, Raney B (1996). Fibromyxoid sarcoma in a four-year-old child: case report and review of the literature. Med Pediatr Oncol.

[28] Rose B, Tamvakopoulos GS, Dulay K, Pollock R, Skinner J, Briggs T, Cannon S (2011). The clinical significance of the FUS-CREB3L2 translocation in low-grade fibromyxoid sarcoma. J Orthop Surg Res.

